# Aqua­(1*H*-benzimidazole-κ*N* ^3^)(pyridine-2,6-dicarboxyl­ato-κ^3^ *O* ^2^,*N*,*O* ^6^)copper(II) 0.75-hydrate

**DOI:** 10.1107/S160053681001353X

**Published:** 2010-04-17

**Authors:** Gui-Ying Dong, Li-Hua Fan, Li-Xia Yang, Islam Ullah Khan

**Affiliations:** a College of Chemical Engineering and Biotechnology, Hebei Polytechnic University, Tangshan 063009, People’s Republic of China; b Materials Chemistry Laboratory, Department of Chemistry, Government College University, Lahore 54000, Pakistan

## Abstract

The title complex, [Cu(C_7_H_3_NO_4_)(C_7_H_6_N_2_)(H_2_O)]·0.75H_2_O, consists of discrete monomeric units. The Cu^II^ atom is coordinated by two carboxyl­ate O atoms and the N atom from a dipicolinate unit and by an N atom from a benzimidazole ligand. The distorted square-pyramidal geometry is completed by a longer axial bond to the O atom of a water mol­ecule. The mol­ecular structure and packing are stabilized by classical O—H⋯O and N—H⋯O hydrogen bonds, also including a disordered crystal water molecule.

## Related literature

For related structures of dipicolinate complexes, see: How *et al.* (1991 ▶). 
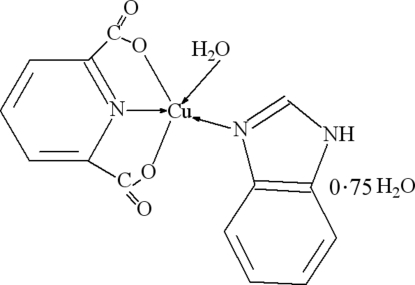


## Experimental

### 

#### Crystal data

[Cu(C_7_H_3_NO_4_)(C_7_H_6_N_2_)(H_2_O)]·0.75H_2_O
*M* *_r_* = 378.32
Monoclinic, 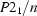

*a* = 8.6388 (17) Å
*b* = 17.692 (4) Å
*c* = 9.783 (2) Å
β = 97.78 (3)°
*V* = 1481.5 (5) Å^3^
*Z* = 4
Mo *K*α radiation
μ = 1.51 mm^−1^
*T* = 295 K
0.26 × 0.24 × 0.18 mm

#### Data collection

Bruker SMART CCD area-detector diffractometer
Absorption correction: multi-scan (*SADABS*; Sheldrick, 1996 ▶) *T*_min_ = 0.677, *T*_max_ = 0.759
12842 measured reflections
2614 independent reflections
2108 reflections with *I* > 2σ(*I*)
*R*_int_ = 0.062

#### Refinement

*R*[*F* ^2^ > 2σ(*F* ^2^)] = 0.049
*wR*(*F* ^2^) = 0.103
*S* = 1.23
2614 reflections
218 parameters
H-atom parameters constrained
Δρ_max_ = 0.63 e Å^−3^
Δρ_min_ = −0.30 e Å^−3^

### 

Data collection: *SMART* (Bruker, 1998 ▶); cell refinement: *SAINT* (Bruker, 1999 ▶); data reduction: *SAINT*; program(s) used to solve structure: *SHELXS97* (Sheldrick, 2008 ▶); program(s) used to refine structure: *SHELXL97* (Sheldrick, 2008 ▶); molecular graphics: *SHELXTL* (Sheldrick, 2008 ▶); software used to prepare material for publication: *SHELXTL*.

## Supplementary Material

Crystal structure: contains datablocks I, global. DOI: 10.1107/S160053681001353X/rk2200sup1.cif

Structure factors: contains datablocks I. DOI: 10.1107/S160053681001353X/rk2200Isup2.hkl

Additional supplementary materials:  crystallographic information; 3D view; checkCIF report

## Figures and Tables

**Table 1:** Hydrogen-bond geometry (Å, °)

*D*—H⋯*A*	*D*—H	H⋯*A*	*D*⋯*A*	*D*—H⋯*A*
O1*W*—H1*A*⋯O2^i^	0.85	1.91	2.740 (4)	164
O2*W*—H2*A*⋯O3	0.85	2.06	2.804 (5)	145
O2*W*—H2*B*⋯O2w^ii^	0.85	2.25	3.037 (9)	154
N3—H3*A*⋯O2^iii^	0.86	1.90	2.756 (4)	175

Symmetry codes: (i) 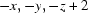; (ii) 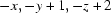; (iii) 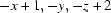.

**Fig. 1.**
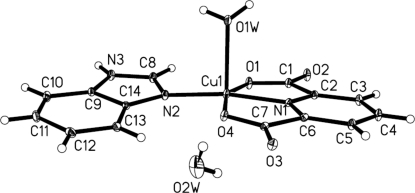
The molecular structure of the title compound, with the atom numbering scheme. The displacement ellipsoids are drawn at 30% probability level. h atoms are presented as a small spheres of arbitrary radius.

## supplementary crystallographic information

### Comment

The dipicolinic acid (pyridine–2,6–dicarboxylic acid) has important biological functions in the organism and commonly coordinate to transition metals by either carboxylate bridges between metal centers, to form polymeric or dimeric complexes or tridentate (O, N, O') chelation to one metal ion. Some Cu(II) dipicolinate complexes with imidazole had been reported (How *et al.*, 1991). Here, we report here the crystal structure of the title compound.

The molecular structure of the title compound, is illustrated in Fig. 1. All the bond lengths and angles are in the normal range (How *et al.*, 1991). The overall molecular structure of title complex, has only independent Cu(II) ion, is five–coordinated by one N and two O atoms from a dipicolinate dianion ligand, one N atom from a benzimidazole molecule and one O atom of a water molecule. In molecular structure, each Cu(II) center exhibits a slightly distorted square pyramidal environment. The intermolecular hydrogen bonds play an important role in the crystal packing and the stability of the complex (Table 1).

### Experimental

Copper(II) hydroxide (98 mg, 1 mmol) was treated with an aqueous solution (10 mL) of dipicoline acid (334 mg, 2 mmol) in a steam bath until the solid disappeared. The solution was then filtered and diluted to approximately 40 mL with water. An methanol solution (10 mL) of benzimidazole (472 mg, 4 mmol) is then added to above solution. The resultant clear–blue solution is warmed on a steam bath for 1 h. The volume is kept constant by periodic addition of water. Then the solution is filtered and allowed to stand at room temperature. Blue crystals suitable for X–ray single diffraction were obtained after 20 days.

### Refinement

All H atoms were positioned geometrically an refined using a riding model with C—H = 0.93Å and *U*_iso_(H) = 1.2*U*_eq_(C) for aromatic H, O—H = 0.85Å and *U*_iso_(H) = 1.5*U*_eq_(O) for the OH group and N—H = 0.86Å and *U*_iso_(H) = 1.2*U*_eq_(N) for the NH group.

### Crystal data

**Table:**

[Cu(C_7_H_3_NO_4_)(C_7_H_6_N_2_)(H_2_O)]·0.75H_2_O	*F*(000) = 770
*M**_r_* = 378.32	*D*_x_ = 1.696 Mg m^−^^3^
Monoclinic, *P*2_1_/*n*	Mo *K*α radiation, λ = 0.71073 Å
Hall symbol: -P 2yn	Cell parameters from 2800 reflections
*a* = 8.6388 (17) Å	θ = 4.4–20.6°
*b* = 17.692 (4) Å	µ = 1.51 mm^−^^1^
*c* = 9.783 (2) Å	*T* = 295 K
β = 97.78 (3)°	Block, blue
*V* = 1481.5 (5) Å^3^	0.26 × 0.24 × 0.18 mm
*Z* = 4	

### Data collection

**Table:**

Bruker SMART CCD area-detector diffractometer	2614 independent reflections
Radiation source: fine–focus sealed tube	2108 reflections with *I* > 2σ(*I*)
graphite	*R*_int_ = 0.062
φ and ω scans	θ_max_ = 25.0°, θ_min_ = 3.1°
Absorption correction: multi-scan (*SADABS*; Sheldrick, 1996)	*h* = −10→10
*T*_min_ = 0.677, *T*_max_ = 0.759	*k* = −21→21
12842 measured reflections	*l* = −11→11

### Refinement

**Table:**

Refinement on *F*^2^	Primary atom site location: structure-invariant direct methods
Least-squares matrix: full	Secondary atom site location: difference Fourier map
*R*[*F*^2^ > 2σ(*F*^2^)] = 0.049	Hydrogen site location: inferred from neighbouring sites
*wR*(*F*^2^) = 0.103	H-atom parameters constrained
*S* = 1.23	*w* = 1/[σ^2^(*F*_o_^2^) + (0.0321*P*)^2^ + 1.3817*P*] where *P* = (*F*_o_^2^ + 2*F*_c_^2^)/3
2614 reflections	(Δ/σ)_max_ < 0.001
218 parameters	Δρ_max_ = 0.63 e Å^−^^3^
0 restraints	Δρ_min_ = −0.29 e Å^−^^3^

### Special details

**Table:**

Geometry. All s.u.'s (except the s.u. in the dihedral angle between two l.s. planes) are estimated using the full covariance matrix. The cell s.u.'s are taken into account individually in the estimation of s.u.'s in distances, angles and torsion angles; correlations between s.u.'s in cell parameters are only used when they are defined by crystal symmetry. An approximate (isotropic) treatment of cell s.u.'s is used for estimating s.u.'s involving l.s. planes.
Refinement. Refinement of F^2^ against ALL reflections. The weighted *R*–factor w*R* and goodness of fit *S* are based on *F*^2^, conventional *R*–factors *R* are based on *F*, with *F* set to zero for negative *F*^2^. The threshold expression of *F*^2^ > 2σ(*F*^2^) is used only for calculating *R*–factors(gt) etc. and is not relevant to the choice of reflections for refinement. *R*–factors based on *F*^2^ are statistically about twice as large as those based on *F*, and *R*–factors based on ALL data will be even larger.

### Fractional atomic coordinates and isotropic or equivalent isotropic displacement parameters (Å^2^)

**Table:**

	*x*	*y*	*z*	*U*_iso_*/*U*_eq_	Occ. (<1)
Cu1	0.21983 (5)	0.15993 (3)	0.91593 (5)	0.03235 (18)	
O1W	0.1635 (3)	0.14651 (17)	1.1317 (3)	0.0490 (8)	
H1A	0.1016	0.1119	1.1510	0.074*	
H1B	0.2504	0.1412	1.1831	0.074*	
O1	0.2325 (3)	0.04517 (15)	0.8889 (3)	0.0428 (8)	
O2	0.0796 (3)	−0.05328 (15)	0.8281 (3)	0.0468 (8)	
O3	−0.0676 (3)	0.33244 (15)	0.8066 (3)	0.0379 (7)	
O4	0.1407 (3)	0.26612 (14)	0.8944 (3)	0.0348 (7)	
N1	0.0128 (3)	0.14008 (17)	0.8315 (3)	0.0272 (7)	
N2	0.4407 (4)	0.17683 (17)	0.9703 (3)	0.0325 (8)	
N3	0.6752 (4)	0.14636 (19)	1.0717 (4)	0.0381 (9)	
H3A	0.7523	0.1188	1.1072	0.046*	
C1	0.1030 (4)	0.0149 (2)	0.8435 (4)	0.0343 (10)	
C2	−0.0305 (4)	0.0691 (2)	0.8052 (4)	0.0290 (9)	
C3	−0.1796 (4)	0.0531 (2)	0.7462 (4)	0.0380 (10)	
H3	−0.2121	0.0035	0.7291	0.046*	
C4	−0.2802 (5)	0.1129 (2)	0.7127 (5)	0.0433 (12)	
H4	−0.3820	0.1035	0.6720	0.052*	
C5	−0.2321 (4)	0.1860 (2)	0.7386 (4)	0.0359 (10)	
H5A	−0.2998	0.2263	0.7154	0.043*	
C6	−0.0812 (4)	0.1984 (2)	0.7998 (4)	0.0279 (9)	
C7	0.0006 (4)	0.2725 (2)	0.8364 (4)	0.0288 (9)	
C8	0.5344 (4)	0.1218 (2)	1.0185 (4)	0.0365 (10)	
H8	0.5055	0.0712	1.0158	0.044*	
C9	0.6754 (4)	0.2233 (2)	1.0597 (4)	0.0314 (9)	
C10	0.7901 (5)	0.2770 (3)	1.1004 (4)	0.0418 (11)	
H10	0.8885	0.2636	1.1444	0.050*	
C11	0.7497 (5)	0.3500 (3)	1.0722 (5)	0.0472 (12)	
H11	0.8222	0.3878	1.0993	0.057*	
C12	0.6032 (5)	0.3706 (3)	1.0040 (5)	0.0448 (11)	
H12	0.5818	0.4213	0.9849	0.054*	
C13	0.4908 (5)	0.3176 (2)	0.9650 (4)	0.0361 (10)	
H13	0.3930	0.3315	0.9204	0.043*	
C14	0.5275 (4)	0.2424 (2)	0.9940 (4)	0.0284 (9)	
O2W	0.0560 (6)	0.4772 (2)	0.8653 (7)	0.100 (2)	0.75
H2A	0.0357	0.4379	0.8162	0.150*	0.75
H2B	0.0004	0.4956	0.9225	0.150*	0.75

### Atomic displacement parameters (Å^2^)

**Table:**

	*U*^11^	*U*^22^	*U*^33^	*U*^12^	*U*^13^	*U*^23^
Cu1	0.0228 (3)	0.0257 (3)	0.0449 (3)	−0.0002 (2)	−0.0085 (2)	−0.0006 (2)
O1W	0.0418 (17)	0.055 (2)	0.0454 (19)	−0.0227 (15)	−0.0100 (14)	0.0104 (15)
O1	0.0249 (15)	0.0278 (16)	0.071 (2)	0.0008 (12)	−0.0099 (15)	−0.0021 (14)
O2	0.0299 (16)	0.0238 (16)	0.082 (2)	−0.0006 (13)	−0.0096 (15)	0.0019 (15)
O3	0.0376 (16)	0.0255 (15)	0.0480 (18)	0.0073 (13)	−0.0035 (13)	0.0005 (13)
O4	0.0279 (15)	0.0263 (15)	0.0464 (18)	0.0003 (12)	−0.0094 (13)	−0.0017 (13)
N1	0.0238 (17)	0.0281 (19)	0.0279 (18)	0.0004 (14)	−0.0031 (14)	0.0008 (14)
N2	0.0261 (17)	0.0265 (19)	0.043 (2)	0.0012 (14)	−0.0033 (15)	−0.0018 (15)
N3	0.0241 (18)	0.035 (2)	0.052 (2)	0.0056 (15)	−0.0041 (16)	0.0006 (17)
C1	0.026 (2)	0.030 (2)	0.045 (3)	−0.0004 (18)	−0.0036 (19)	0.0017 (19)
C2	0.025 (2)	0.026 (2)	0.035 (2)	0.0017 (17)	0.0005 (17)	0.0020 (17)
C3	0.026 (2)	0.031 (2)	0.054 (3)	−0.0034 (18)	−0.004 (2)	−0.002 (2)
C4	0.022 (2)	0.042 (3)	0.062 (3)	0.0002 (19)	−0.009 (2)	0.000 (2)
C5	0.024 (2)	0.031 (2)	0.050 (3)	0.0070 (18)	−0.0034 (19)	0.0034 (19)
C6	0.028 (2)	0.028 (2)	0.027 (2)	0.0034 (18)	0.0009 (17)	0.0007 (17)
C7	0.031 (2)	0.031 (2)	0.024 (2)	0.0020 (18)	0.0024 (18)	−0.0017 (17)
C8	0.027 (2)	0.031 (2)	0.048 (3)	0.0026 (19)	−0.005 (2)	−0.001 (2)
C9	0.024 (2)	0.036 (2)	0.033 (2)	−0.0030 (18)	0.0014 (18)	−0.0016 (18)
C10	0.026 (2)	0.051 (3)	0.048 (3)	−0.005 (2)	0.002 (2)	−0.007 (2)
C11	0.036 (3)	0.051 (3)	0.055 (3)	−0.019 (2)	0.008 (2)	−0.008 (2)
C12	0.053 (3)	0.033 (2)	0.050 (3)	−0.007 (2)	0.012 (2)	0.003 (2)
C13	0.032 (2)	0.035 (2)	0.041 (3)	−0.0020 (19)	0.0032 (19)	0.0050 (19)
C14	0.025 (2)	0.031 (2)	0.030 (2)	−0.0026 (17)	0.0050 (17)	−0.0011 (17)
O2W	0.102 (4)	0.026 (3)	0.169 (6)	−0.016 (3)	0.001 (4)	−0.020 (3)

### Geometric parameters (Å, °)

**Table:**

Cu1—N1	1.898 (3)	C3—C4	1.380 (5)
Cu1—N2	1.934 (3)	C3—H3	0.9300
Cu1—O4	2.001 (3)	C4—C5	1.372 (6)
Cu1—O1	2.052 (3)	C4—H4	0.9300
Cu1—O1W	2.242 (3)	C5—C6	1.377 (5)
O1W—H1A	0.8500	C5—H5A	0.9300
O1W—H1B	0.8500	C6—C7	1.509 (5)
O1—C1	1.265 (4)	C8—H8	0.9300
O2—C1	1.229 (5)	C9—C14	1.392 (5)
O3—C7	1.229 (4)	C9—C10	1.391 (5)
O4—C7	1.270 (4)	C10—C11	1.358 (6)
N1—C6	1.324 (5)	C10—H10	0.9300
N1—C2	1.325 (5)	C11—C12	1.396 (6)
N2—C8	1.312 (5)	C11—H11	0.9300
N2—C14	1.384 (5)	C12—C13	1.366 (6)
N3—C8	1.329 (5)	C12—H12	0.9300
N3—C9	1.366 (5)	C13—C14	1.389 (5)
N3—H3A	0.8600	C13—H13	0.9300
C1—C2	1.507 (5)	O2W—H2A	0.8500
C2—C3	1.368 (5)	O2W—H2B	0.8500
			
N1—Cu1—N2	169.98 (14)	C5—C4—H4	119.5
N1—Cu1—O4	80.76 (12)	C3—C4—H4	119.5
N2—Cu1—O4	101.21 (12)	C4—C5—C6	118.4 (4)
N1—Cu1—O1	79.88 (12)	C4—C5—H5A	120.8
N2—Cu1—O1	96.93 (12)	C6—C5—H5A	120.8
O4—Cu1—O1	159.86 (10)	N1—C6—C5	119.5 (4)
N1—Cu1—O1W	94.52 (12)	N1—C6—C7	111.7 (3)
N2—Cu1—O1W	95.10 (13)	C5—C6—C7	128.8 (4)
O4—Cu1—O1W	94.81 (11)	O3—C7—O4	125.4 (4)
O1—Cu1—O1W	92.20 (12)	O3—C7—C6	120.0 (3)
Cu1—O1W—H1A	120.5	O4—C7—C6	114.6 (3)
Cu1—O1W—H1B	106.3	N2—C8—N3	112.7 (4)
H1A—O1W—H1B	108.7	N2—C8—H8	123.7
C1—O1—Cu1	113.8 (2)	N3—C8—H8	123.7
C7—O4—Cu1	114.9 (2)	N3—C9—C14	105.7 (3)
C6—N1—C2	123.0 (3)	N3—C9—C10	131.7 (4)
C6—N1—Cu1	118.0 (3)	C14—C9—C10	122.7 (4)
C2—N1—Cu1	119.0 (3)	C11—C10—C9	116.0 (4)
C8—N2—C14	105.5 (3)	C11—C10—H10	122.0
C8—N2—Cu1	121.5 (3)	C9—C10—H10	122.0
C14—N2—Cu1	131.9 (3)	C10—C11—C12	122.4 (4)
C8—N3—C9	107.7 (3)	C10—C11—H11	118.8
C8—N3—H3A	126.2	C12—C11—H11	118.8
C9—N3—H3A	126.2	C13—C12—C11	121.3 (4)
O2—C1—O1	125.5 (4)	C13—C12—H12	119.3
O2—C1—C2	119.1 (3)	C11—C12—H12	119.3
O1—C1—C2	115.4 (3)	C12—C13—C14	117.8 (4)
N1—C2—C3	120.2 (4)	C12—C13—H13	121.1
N1—C2—C1	111.6 (3)	C14—C13—H13	121.1
C3—C2—C1	128.2 (4)	N2—C14—C9	108.4 (3)
C2—C3—C4	117.9 (4)	N2—C14—C13	131.8 (4)
C2—C3—H3	121.1	C9—C14—C13	119.8 (4)
C4—C3—H3	121.1	H2A—O2W—H2B	126.4
C5—C4—C3	120.9 (4)		

### Hydrogen-bond geometry (Å, °)

**Table:**

*D*—H···*A*	*D*—H	H···*A*	*D*···*A*	*D*—H···*A*
O1W—H1A···O2^i^	0.85	1.91	2.740 (4)	164
O2W—H2A···O3	0.85	2.06	2.804 (5)	145
O2W—H2B···O2w^ii^	0.85	2.25	3.037 (9)	154
N3—H3A···O2^iii^	0.86	1.90	2.756 (4)	175

Symmetry codes: (i) −*x*, −*y*, −*z*+2;  (ii) −*x*, −*y*+1, −*z*+2; (iii) −*x*+1, −*y*, −*z*+2.
